# HSV Usurps Eukaryotic Initiation Factor 3 Subunit M for Viral Protein Translation: Novel Prevention Target

**DOI:** 10.1371/journal.pone.0011829

**Published:** 2010-07-27

**Authors:** Natalia Cheshenko, Janie B. Trepanier, Theodore J. Segarra, A. Oveta Fuller, Betsy C. Herold

**Affiliations:** 1 Departments of Pediatrics and Microbiology-Immunology, Albert Einstein College of Medicine, Bronx, New York, United States of America; 2 Department of Microbiology and Immunology, University of Michigan, Ann Arbor, Michigan, United States of America; University of Minnesota, United States of America

## Abstract

Prevention of genital herpes is a global health priority. B5, a recently identified ubiquitous human protein, was proposed as a candidate HSV entry receptor. The current studies explored its role in HSV infection. Viral plaque formation was reduced by ∼90% in human cells transfected with small interfering RNA targeting B5 or nectin-1, an established entry receptor. However, the mechanisms were distinct. Silencing of nectin-1 prevented intracellular delivery of viral capsids, nuclear transport of a viral tegument protein, and release of calcium stores required for entry. In contrast, B5 silencing had no effect on these markers of entry, but inhibited viral protein translation. Specifically, viral immediate early genes, ICP0 and ICP4, were transcribed, polyadenylated and transported from the nucleus to the cytoplasm, but the viral transcripts did not associate with ribosomes or polysomes in B5-silenced cells. In contrast, immediate early gene viral transcripts were detected in polysome fractions isolated from control cells. These findings are consistent with sequencing studies demonstrating that B5 is eukaryotic initiation factor 3 subunit m (eIF3m). Although B5 silencing altered the polysome profile of cells, silencing had little effect on cellular RNA or protein expression and was not cytotoxic, suggesting that this subunit is not essential for host cellular protein synthesis. Together these results demonstrate that B5 plays a major role in the initiation of HSV protein translation and could provide a novel target for strategies to prevent primary and recurrent herpetic disease.

## Introduction

Herpes simplex viruses (HSV) are the leading cause of genital herpes worldwide, the most common infection associated with neonatal encephalitis, and a major co-factor for HIV infection, thus underscoring the urgency to develop novel prevention strategies [1]. Notably, the epidemiology of genital herpes may be changing as recent studies indicate that HSV-1 accounts for a significant proportion of new infections, particularly in the developed world [2], [3]. Identifying new approaches to prevent infection by both serotypes requires an understanding of the pathways required for the establishment of primary and recurrent infection and the cellular factors usurped by the viruses to promote infection.

Preventing HSV entry has proved difficult, reflecting the complexity of this process, which involves interactions between several viral envelope glycoproteins and cellular receptors and activation of calcium signaling pathways. Both serotypes (HSV-1 and HSV-2) initiate infection by binding to heparan sulfate moieties on syndecan proteoglycans [4], [5], [6], [7]. Glycoprotein D (gD) then engages one of several entry receptors, most commonly nectin-1 or herpes virus entry mediator (HVEM) [8], [9]. Studies with human epithelial cells indicate that these viral-cell interactions trigger the release of calcium (Ca^2+^) near the plasma membrane, which is followed by activation of the inositol triphosphate receptor, resulting in the rapid release of endoplasmic reticulum (ER) Ca^2+^ stores [4], [10]. This release of ER stores requires the concerted activities of glycoproteins B, D, and hetero-oligomers of H and L and blockade of the Ca^2+^ response prevents viral entry.

Recent work suggested that another cellular protein also may play a role in HSV entry. Porcine renal epithelial cells, which are naturally resistant to HSV entry, were rendered fully susceptible following transfection with a cDNA encoding human *hfl-B5* [11], [12], [13], [14]. The B5 protein was found to be ubiquitously expressed on multiple human cell lines and a synthetic 30-mer peptide containing the sequence found in the C-terminus of B5 inhibited HSV infection at a step following viral attachment [14]. Recent genetic studies demonstrate that B5 is identical to the sequence that encodes for subunit m of eukaryotic initiation factor 3 (eIF3m) [15]. Building from these observations, we sought to further explore the role B5 (eiF3m) plays in HSV infection of human cells and whether it could provide a target for the development of novel prevention strategies.

## Results

### Silencing of B5 inhibits HSV infection

**Figure 1:**
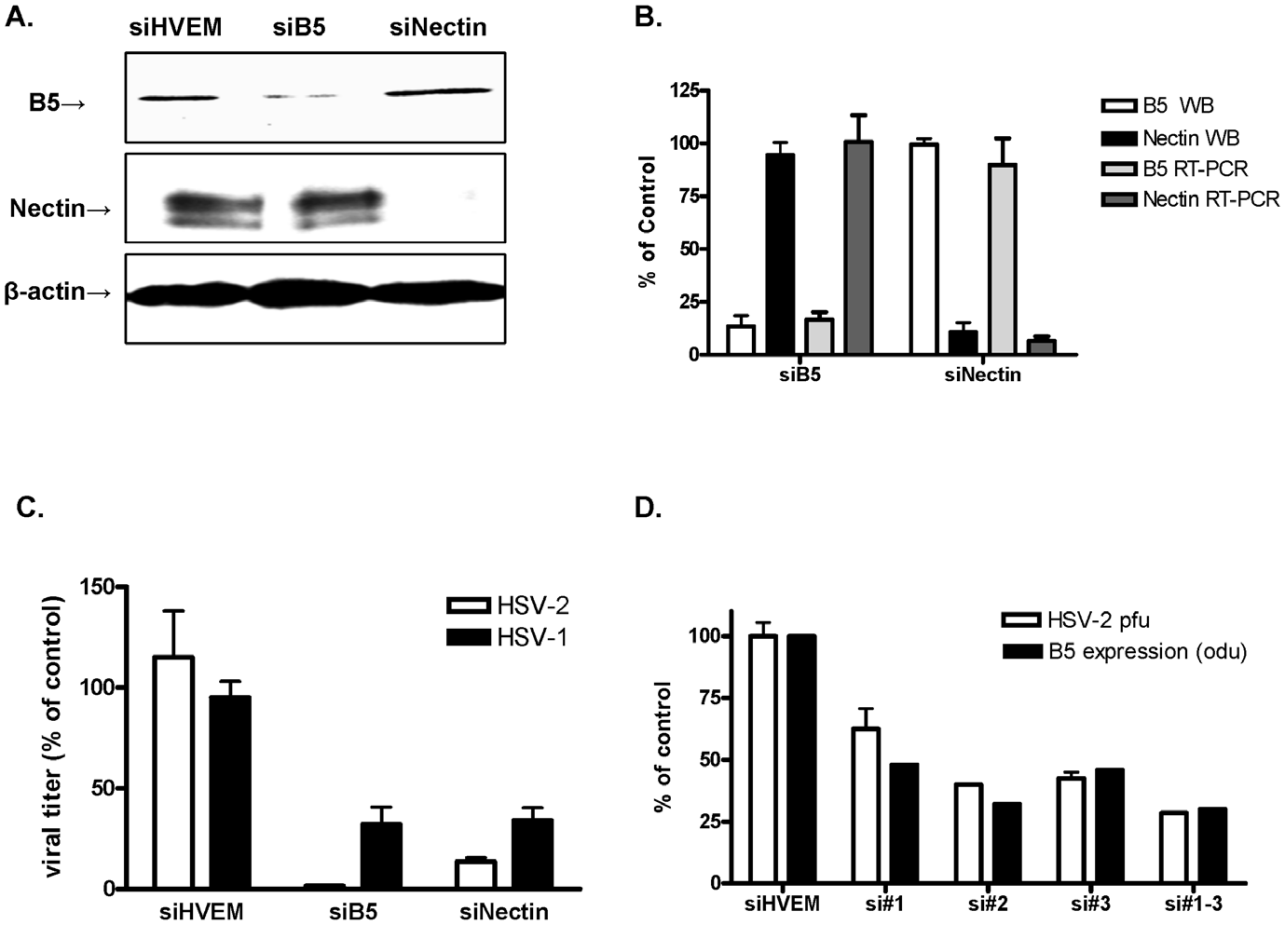
Silencing of B5 and nectin-1 reduces HSV infection. (A). CaSki cells were transfected with siHVEM (control), B5 or nectin-1-specific pools of siRNA and 48 h post-transfection, immunoblots of cell lysates were prepared and probed with anti-B5 polyclonal Ab or anti-nectin mAb and then stripped and probed for β-actin expression. (B) Western blots were scanned for protein expression (odu). In parallel studies, RNA was extracted from transfected cells and analyzed by qRT-PCR for expression of B5 and nectin. Results are presented as protein or RNA expression as a percentage of cells transfected with siHVEM and are means ± sd obtained from 3 different experiments. (C) The transfected cells were infected with HSV-2(G) or HSV-1(KOS) (48 h post-transfection) and the ability to support viral plaque formation determined by counting plaques after 48 h incubation by immunostaining. Results are presented as pfu/well as a percentage of pfu/well formed in cells transfected with non-specific control siRNA and are means + sd from 2 independent experiments conducted in quadruplicate. (D) Cells were transfected independently with each siB5, with the pool of three siRNAs or with siHVEM (control) and 48 h post-transfection immunoblots of cell lysates were prepared and probed with anti-B5 polyclonal Ab and β-actin and relative expression determined after scanning the blots. Results are presented as odu relative to cells transfected with siHVEM. In parallel, the transfected cells were infected with HSV-2(G) and the ability to support viral plaque formation determined by counting plaques after 48 h incubation by immunostaining. Results are presented as pfu/well as percentage of pfu/well formed in cells transfected with siHVEM and are means ± sd from a representative experiment conducted in duplicate.

CaSki (human cervical epithelial) cells were transfected with siRNA targeting B5, nectin-1, an established entry co-receptor, or as a control, HVEM, an alternative co-receptor that is not expressed at detectable levels on CaSki cells [4]. Silencing resulted in reductions of 80–95% in protein and RNA expression by Western blot and quantitative real time PCR (qRT-PCR), respectively, compared to cells transfected with siHVEM (Figs 1A and B) or with a non-specific control siRNA (not shown). Silencing was specific, as transfection with siB5 had no impact on nectin expression and, conversely, transfection with siNectin had no effect on B5 expression. To determine whether silencing impacted HSV infection, CaSki cells were transfected with siB5, siNectin, or siHVEM. The transfected cells were infected with HSV-2(G) or HSV-1(KOS) 48 h post-transfection and viral plaques were quantified. Transfection with siB5 or siNectin reduced HSV-2 plaque formation by 98.6±0.8 and 86.5±3.5% respectively and HSV-1 plaque formation by 68±15 and 66±11%, respectively, relative to cells transfected with a non-specific control siRNA, whereas silencing of HVEM had little or no effect (Fig. 1C). Transfection with each individual siRNA targeting B5 also resulted in a reduction in B5 protein expression and concomitant inhibition of HSV-2 plaque formation, although siB5#1 was the least efficient (Fig. 1D). Importantly, transfection with siB5 was not cytotoxic and did not trigger any significant interferon response (not shown). Together, these findings support a role for B5 in HSV infection.

### B5 is not required for HSV entry

**Figure 2:**
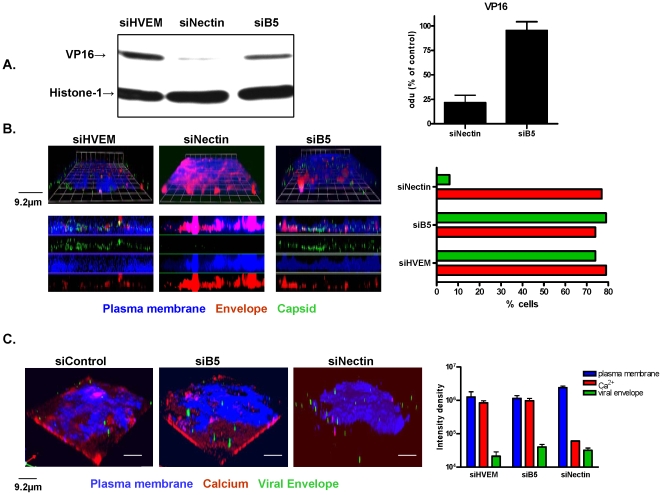
Silencing of nectin, but not B5, prevents HSV entry. (A). CaSki cells were transfected with siNectin, siHVEM or siB5 and then 48 h later, synchronously infected with HSV-2(G) and 45 min after the temperature shift to 37°C, nuclear extracts were prepared and analyzed for presence of viral VP16 by Western blot; expression of histone-1 was included as a control. The bar graph depicts expression of VP16 relative to histone-1; results are representative of three independent experiments. (B). Cells were transfected with siRNA and then 48 h post-transfection, infected with DiI-envelope labeled KVP26GFP and 15 min later, fixed and stained for plasma membrane (blue) and analyzed by confocal microscopy. Images are representative of 3 independent experiments: 100 cells were counted and the bar graph depicts percentage of cells expressing bound virus (red envelope) and viral capsids (green). (C). Representative 3-dimensional images obtained by live confocal microscopy of transfected cells, stained for plasma membrane (blue), loaded with Calcium Crimson and then synchronously infected with DiO-envelope labeled HSV-2(G) (green). Images shown were obtained 10 min after being placed in a 37°C temperature-controlled chamber. The bar graph on the right depicts relative intensity obtained from scanning 10 cells per field of vision from two independent experiments.

Several parallel strategies were adopted to determine whether B5 silencing interfered with viral entry. VP16 is a virion tegument protein delivered to the nucleus following HSV entry and thus serves as a surrogate marker of viral entry. CaSki cells were transfected with nectin-1, control, or B5 specific siRNA, and then 48 h later, synchronously infected with HSV-2(G) (moi 1 pfu/cell). Forty-five minutes post-shift of the temperature to 37°C, cells were harvested and nuclear extracts were prepared and evaluated for the presence of VP16 by preparing Western blots; expression of nuclear protein histone-1 was included as a control. As anticipated, silencing of nectin-1 significantly blocked the nuclear transport of VP16 by 78±15% (p<0.01, t-test), consistent with a block to viral entry, whereas silencing of B5 had no impact on VP16 nuclear delivery relative to cells transfected with control siRNA (Fig. 2A).

We also applied confocal microscopy to directly monitor the intracellular delivery of viral capsids. CaSki cells were transfected with each of the siRNAs and 48 h later, synchronously infected (moi 5 pfu/cell) with purified KVP26GFP, an HSV-1 variant expressing a VP26–GFP fusion capsid protein, for which the viral envelope was also labeled with a fluorescent long-chain carbocyanine dye, DiI [4], [16]. This enabled discrimination between bound viral envelopes (red) and intracellularly delivered capsids (green). Notably, as previously reported, the GFP fluorescence in the capsid is only visualized after viral de-envelopment when the viral envelope is labeled with DiI [4]. The cells were stained with EZ-Link Sulfo-NHS-Biotin to detect plasma membranes (blue) before infection and were fixed 15 minutes after entry. Bound viral envelopes (red) were readily detected in all of the transfectants. However, while viral capsids (green) were observed in the majority of cells transfected with siHVEM (control) or B5-specific siRNA, they were seen in fewer than 6% of cells transfected with nectin-1 siRNA (Fig. 2B).

The block to entry in response to nectin-1, but not B5 silencing, was further confirmed by comparing the intracellular Ca^2+^ response to HSV-2(G) (moi 5) by live confocal microscopy [4]. We previously reported that HSV triggers the release of intracellular Ca^2+^, which promotes viral entry. Viral envelopes (DiO labeled, green) were readily detected bound to the plasma membrane (blue) of cells 10 minutes after the temperature reached 37°C, but release of intracellular Ca^2+^ (crimson) was only observed in the control or siB5 transfected cells, not the siNectin-1 transfectants (Fig. 2C). No intracellular Ca^2+^ response was detected in cells transfected with siNectin as late as 30 minutes following infection (not shown). Additionally, phosphorylation of focal adhesion kinase, a cellular response to viral entry that promotes transport of the viral capsids to the nuclear pore, was observed following HSV infection of cells transfected with siB5, but not cells transfected with siNectin (not shown) [17]. Together, these findings demonstrate that B5 contributes to HSV infection at a step downstream of viral entry and nuclear transport.

### B5 plays key role in the translation of HSV proteins

**Figure 3:**
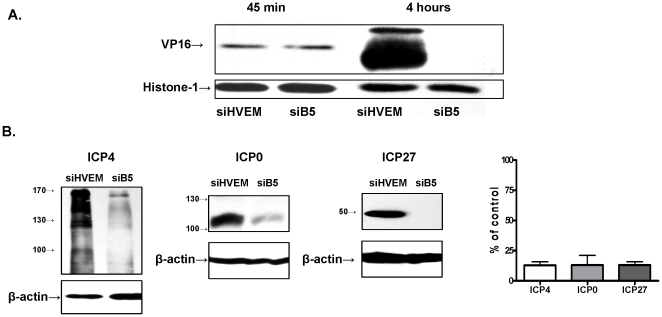
Silencing of B5 blocks viral protein expression. (A). CaSki cells were transfected with siB5 or siHVEM and then 48 h later, synchronously infected with HSV-2(G) and nuclear extracts harvested at the indicated times and evaluated for VP16. Blots are representative of results obtained in 3 independent experiments. (B). Transfected cells were infected with HSV-1(KOS) and 4 h pi, cellular lysates harvested and analyzed for the expression of ICP4, ICP0 and ICP27. Blots were scanned and the relative expression of viral proteins by odu is presented as a percentage of expression in cells transfected with siHVEM obtained in 3 independent experiments (right).

The finding that the sequence for B5 is identical to that of eIF3M [15] suggested that B5 might participate in the initiation of viral protein translation. To address this possibility, CaSki cells were again transfected with siB5 or siHVEM as a control, infected (moi 1 pfu/cell) with HSV-2(G) 48 h post-transfection, and then nuclear or whole cell lysates prepared at different times post-infection (pi) were evaluated for viral protein expression by Western blots. Incoming VP16 was readily detected in nuclear extracts prepared 45 min post-HSV-2 infection in cells transfected with siHVEM (control) and siB5, reflecting the absence of any block to viral entry or nuclear transport. However, 4 h pi, little or no newly synthesized VP16 could be detected in cells transfected with siB5 compared to cells transfected with siHVEM (Fig. 3A). Comparable results were obtained for other viral proteins including thymidine kinase and gB (not shown). A marked reduction in expression of viral immediate early genes (ICP0, ICP4 and ICP27) was observed in whole cell lysates harvested 4 h pi from cells infected with HSV-1(KOS) (Fig. 3B).

**Figure 4:**
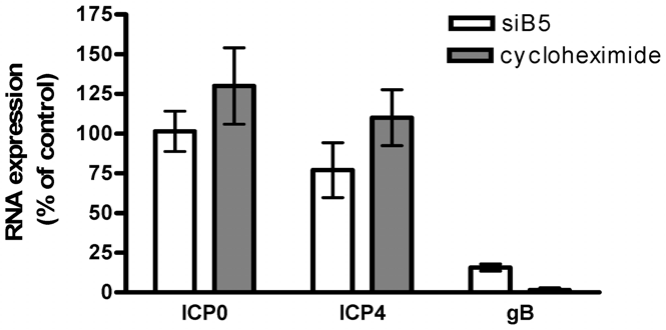
Silencing of B5 blocks translation, but not transcription, of viral proteins. Cells were transfected with siHVEM or siB5 and then infected with HSV-2(G) and 4 h pi, RNA was extracted and analyzed by qRT-PCR for viral gene expression. RNA was also extracted from non-transfected CaSki cells that had been infected with HSV-2(G) in the presence of cycloheximide. Results are presented as RNA expressed in siB5 transfected cells or in cells infected in the presence of cycloheximide as a percentage of expression in siHVEM transfected cells and are mean ± sd obtained from 3 independent experiments.

To determine whether the reduction in protein expression reflects a block in transcription or translation, transfected cells were infected (moi 1 pfu/cell) with HSV-2(G) and then RNA was isolated at different times pi and analyzed for the expression of ICP0, ICP4 or gB by qRT-PCR. As an additional control, cells were infected with virus and then treated with 100µg of cycloheximide, an inhibitor of protein synthesis. Neither transfection with siB5 nor treatment with cycloheximide prevented transcription of ICP0 or ICP4 (Fig. 4). However there was a marked reduction in gB transcripts, which presumably reflects the requirement for ICP0 and ICP4 proteins, which are transcriptional transactivators of late viral gene expression [18], [19]. These results suggest that siB5 does not block immediate early gene expression at transcription, but blocks the translation of these viral proteins.

### Cross-linking of protein-RNA complexes indicates that B5 interacts with viral mRNA

**Figure 5:**
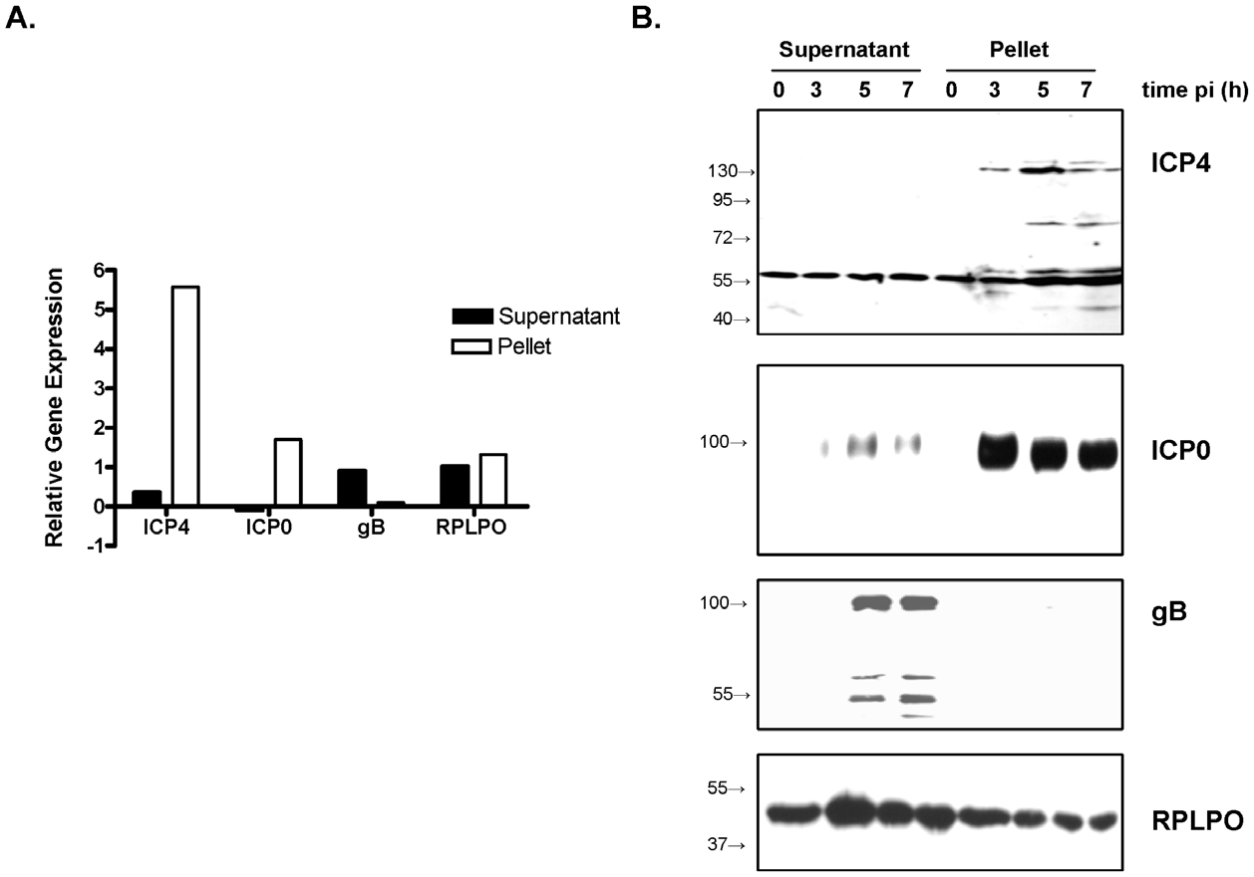
Viral RNA and immediate early proteins associate with B5 in RNA-protein complexes. (A) Cells were infected with HSV-2(G) and 3, 5, or 7 h pi, the cells were treated with 0.04% of methanol-free formaldehyde to cross-link RNA-protein complexes. The complexes were immunoprecipitated with a polyclonal rabbit anti-B5. and RNA was extracted from the supernatant and pellet and analyzed for the expression of viral transcripts and RPLPO. Results are shown for the time pi at which peak expression of viral transcripts was detected (3 h for ICP4, 5 h for ICP0 and 7 h for gB) and are presented as the ratio of viral transcript/RPLPO in each fraction to the viral transcipts/RPLPO in the total RNA-protein complex from infected cells prior to immunoprecipitation (input). Expression of RPLPO is presented as the ratio of RPLPO in each fraction to RPLPO in the total RNA-protein complex from uninfected cells prior to immunoprecipitation (input). (B) The supernatant and pellet from the immunoprecipitated complexes isolated at 0, 3, 5, and 7 h pi were evaluated for the expression of ICP4 (HSV-2), gB (HSV-2), ICP0 (HSV-1) or RPLPO by Western blots. Blots are representative of results obtained in at least 3 independent experiments.

To explore whether B5 interacts directly with viral mRNA, cells were infected with HSV-2(G) (2 pfu/cell) and 3, 5 or 7 h pi, the cells were treated with formaldehyde to cross-link RNA-protein complexes, and then the complexes were immunoprecipitated with a polyclonal antibody to B5. Western blots confirmed that B5 was found exclusively in the pellet (not shown). RNA was extracted from both the supernatant and pellet and evaluated for the presence of viral transcripts and RPLPO by qRT-PCR. The ICP4 and ICP0 transcripts were detected almost exclusively in the B5-immunoprecipitated pellet with peak expression detected at 3 and 5 hours pi, respectively. In contrast, gB transcripts were primarily detected in the supernatant with peak expression 7 h pi and RPLPO transcripts, a ribosomal host cellular gene, were detected in both the supernatant and pellet (Fig. 5A).

In parallel studies, the complexes were subjected to Western blotting with antibodies to ICP4 or gB for HSV-2 and ICP0 for HSV-1; ICP0 was not evaluated for HSV-2 as the antibody is serotype specific. ICP4 and ICP0 were detected in the pellet as early as 3 h pi, whereas gB was detected primarily in the supernatant 5 and 7 h pi and RPLPO was detected in both the supernatant and pellet (Fig. 5B). These findings are consistent with the qRT-PCR results (Fig. 5A) and suggest that B5 interacts with immediate early gene transcripts to promote their translation.

### B5 is required for translation of viral ICP0 and ICP4

**Figure 6:**
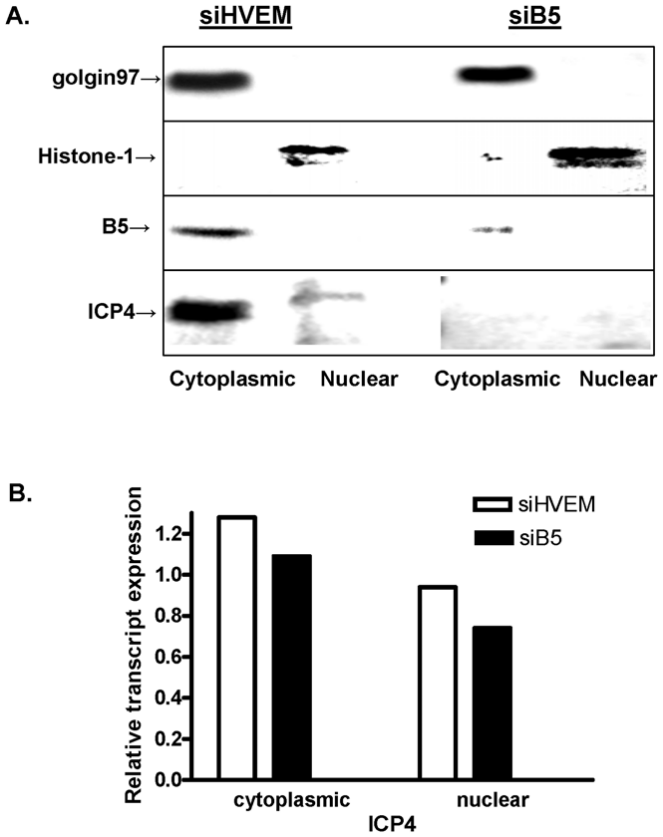
B5 silencing does not block polyadenylation or nuclear export of RNA. (A) Transfected cells were infected with HSV-2(G) (moi 3 pfu/cell) and 3 h pi, cells were harvested and nuclear and cytoplasmic fractions isolated and Western blots were prepared and probed for golgin97, histone-1, B5 and ICP4. (B) Polyadenylated mRNA was isolated from the total RNA and from the cytoplasmic and nuclear fractions and then analyzed for ICP4 by qRT-PCR. Results are presented as the ratio of ICP4/RPLPO in each fraction (cytoplasmic or nuclear) to ICP4/RPLPO in the unfractionated total polyA RNA and are representative of 3 independent experiments.

The observation that silencing of B5 led to a reduction in HSV immediate early proteins without affecting mRNA expression suggests that B5 is required for translation of the viral immediate early proteins. Specifically, silencing could interfere with mRNA processing, such as polyadenylation, nuclear export, the association of RNA with ribosomal subunits or polysomes and/or translation of the mRNA to protein. To address these possibilities, additional experiments were conducted. Transfected cells were infected with HSV-2(G) (3 pfu/cell) and 3 h pi, cells were harvested and nuclear and cytoplasmic fractions isolated. Golgin97 and histone-1 were detected in the cytoplasmic and nuclear fractions, respectively, confirming the purity of the fractionation. B5 and ICP4 were detected in the cytoplasm of cells transfected with control siHVEM, but expression was markedly reduced or absent in cells transfected with siB5 (Fig. 6A). Polyadenylated mRNA was purified from the total isolated RNA and from the cytoplasmic and nuclear fractions and visualized in a formaldehyde denaturing gel. No differences in polyadenylated RNA were detected (not shown) and similar proportions of ICP4 polyadenylated mRNA were detected by qRT-PCR in both the nuclear and cytoplasmic fractions in cells transfected with siHVEM or siB5 (Fig. 6B). Together, these results indicate that B5 silencing had no deleterious impact on polyadenylation or nuclear export of RNA.

**Figure 7:**
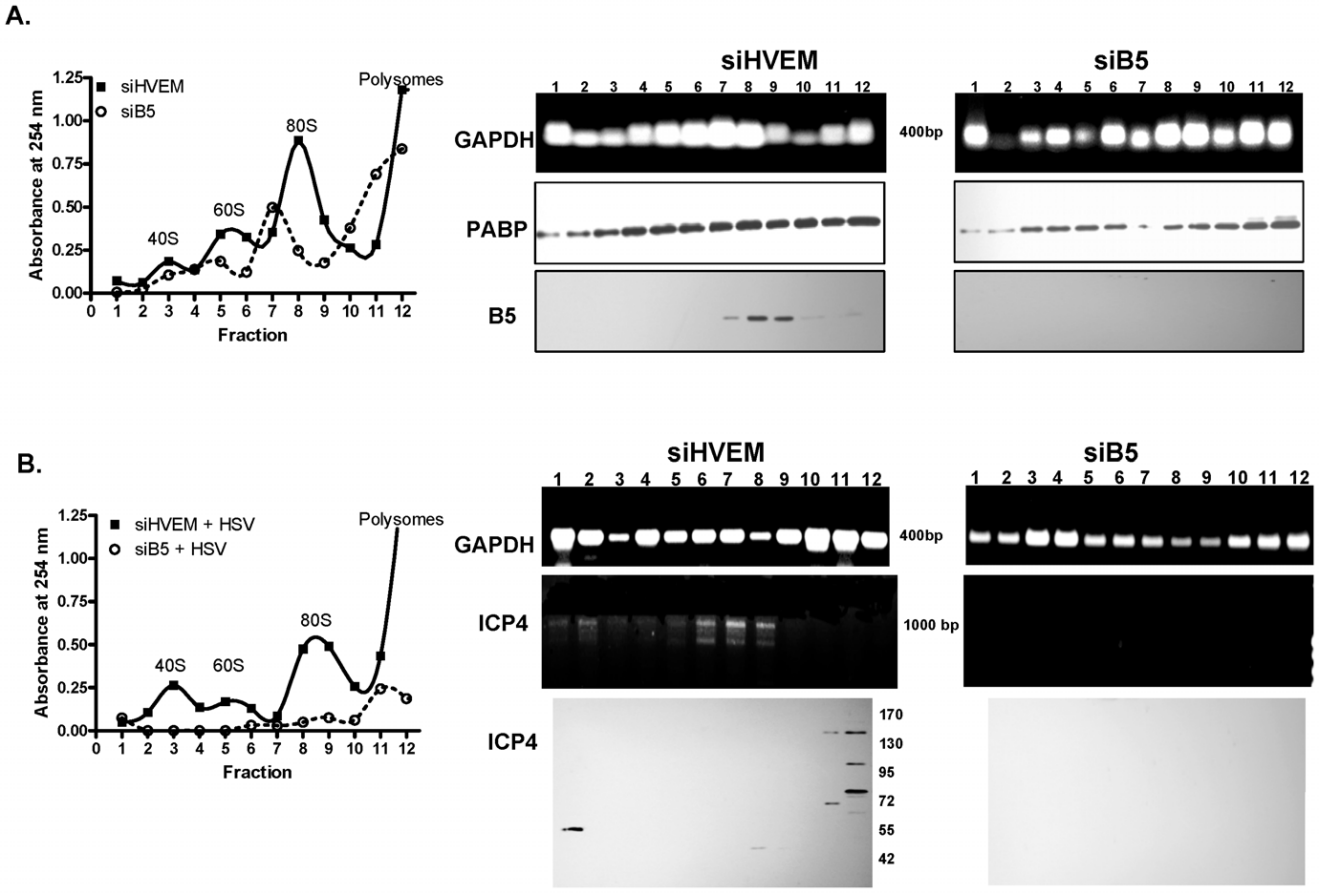
Silencing of B5 prevents HSV ICP4 transcripts from associating with ribosomes. (A). Cytoplasmic extracts of CaSki cells transfected 48 h earlier with siHVEM or siB5 were fractionated on 10% to 50% sucrose gradients and the UV absorbance at 254 nm of each fraction (bottom to top of the sucrose gradient) measured; the relative positions of 40S and 60S ribosomal subunits, 80S monosomes and polysomes are indicated (left). The RNA from each fraction was analyzed for GAPDH expression by reverse transcription and PCR. Alternatively, fractions were analyzed for expression of PABP and B5 by Western blot (right). (B). Cytoplasmic extracts from HSV-2 infected cells that had been transfected 48 h earlier with siHVEM or siB5 were fractionated on 10% to 50% sucrose gradients and the UV absorbance at 254 nm of each fraction measured (left). The RNA from each fraction was analyzed for GAPDH or ICP4 expression by reverse transcription and PCR and the expression of ICP4 protein by Western blot (right).

To determine if silencing of B5 impacted the ability of viral transcripts to associate with ribosomal subunits or polysomes, polysome fractions were isolated from cells that had been transfected with siHVEM (control) or with siB5 by centrifugation through 10–50% sucrose gradients. The UV absorbance pattern of fractions derived from cells that had been transfected with siB5 differed from the pattern observed in siHVEM transfected cells, consistent with B5 playing a role in the formation of ribosomal subunits (Fig. 7A, left). RNA was isolated from each fraction and analyzed for expression of GAPDH by reverse-transcription and PCR and, in parallel studies, the proteins in each fraction were analyzed by Western blot for cytolasmic poly(A)-binding protein 1 (PABP), which is a general translation factor for mRNA, and for B5. GAPDH and PABP were present across the gradient from both siHVEM and siB5 transfected cells, although modest differences in relative amounts expressed in various fractions were observed (Fig. 7A, right). B5 protein was detected predominantly in fractions 7–9 of the siHVEM control cells, coinciding with the 80S ribosomal subunit, and was absent in the siB5 transfected cells.

Parallel studies were conducted following infection with HSV-2. Consistent with published studies, the gradient profile following viral infection in the control (siHVEM) cells showed reduced levels of the ribosomal subunits compared to uninfected cells, presumably reflecting viral-induced shutoff of host protein synthesis (Fig. 7B, left) [20]. The gradient profile was further altered in the siB5-transfected cells, possibly reflecting the combined effects of silencing and viral infection. GAPDH was again distributed across the gradient in fractions isolated from infected cells transfected with either siHVEM or siB5, but ICP4 transcripts were only detected in the fractions isolated from the control (siHVEM) cells and were found primarily in fractions 6–8, which coincides with the fractions containing B5 protein (Fig. 7B, right). Similarly, Western blots demonstrated that ICP4 polypeptides could only be detected in the fractions isolated from control, but not siB5 transfectants (Fig. 7B, right)). Taken together, these results suggest that in the absence of B5, ICP4 transcripts fail to associate with ribosomal subunits, resulting in a block in translation.

### B5 silencing has a greater impact on HSV than VSV or HIV infection

**Figure 8:**
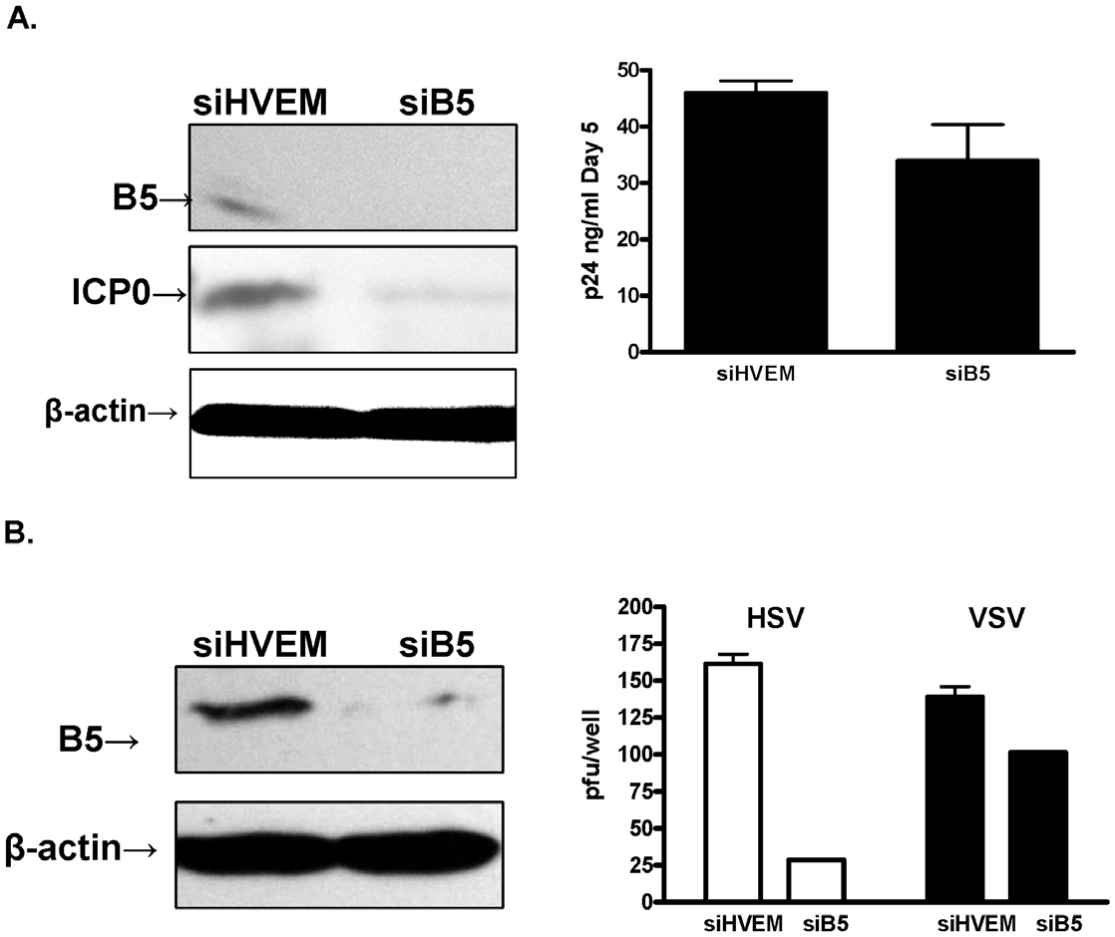
B5 silencing has more modest effects on HIV or VSV infection. (A) Jurkat-TAT-CCR5 cells were transfected with siHVEM (control) or B5 specific siRNA and 48 h post-transfection, immunoblots of cell lysates were prepared and probed with anti-B5 polyclonal Ab (upper panel). Transfected cells were infected with HSV-1(KOS) and 4 h pi, cell lysates prepared and analyzed for ICP0 expression (middle panel) and β-actin as a loading control (lower panel). In parallel, the transfected cells were infected with HIV-1(BaL) and culture supernatants harvested 5 days pi and analyzed for p24 expression (right). (B) CaSki cells were transfected with HVEM or B5 specific siRNA and 48 h post-transfection immunoblots of cell lysates were prepared and probed with anti-B5 polyclonal Ab (upper) and β-actin as a loading control (lower panel). The transfected cells were infected with HSV-2(G) or VSV and plaques counted 48 h pi; results are means ± sd obtained from two independent experiments conducted in duplicate.

Studies were extended to other cell types and other viruses to explore whether B5 also played a major role in protein synthesis and infection. Silencing of B5 inhibited HSV infection of HaCAT cells (human keratinocyte) and murine fibroblasts (not shown). Moreover, there was a reduction in ICP0 protein expression in siB5 transfected Jurkat-TAT-CCR5 cells following HSV-1 infection (Fig. 8A, left). In contrast, silencing of B5 in these cells had a non-significant impact on HIV infection as measured by p24 in culture supernatants (p = 0.2) (Fig. 8A, right). Transfection of CaSki cells with siB5 had more modest effects on VSV (p = 0.03) relative to the inhibitory effects on HSV (p = 0.002) (Fig. 8B).

### Silencing of eIF2α, but not B5, is cytotoxic

**Figure 9:**
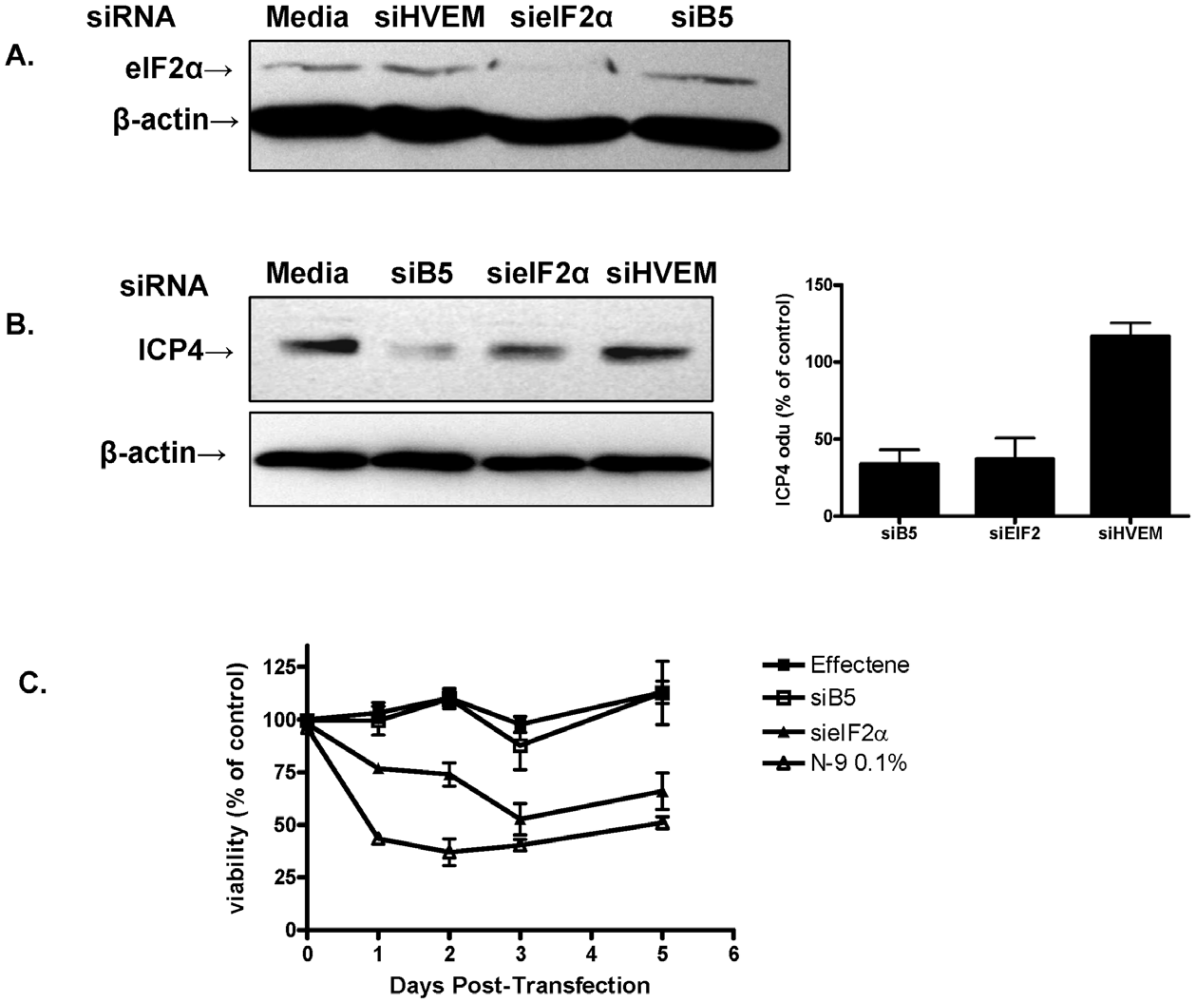
Silencing of eIF2α, but not B5, is cytotoxic. (A) Cells were transfected with HVEM, eIF2α or B5 specific siRNA or no transfection (media) and 48h later, evaluated for the expression of eIF2α and β-actin (control) by Western blot; blots are representative of 2 independent experiments. (B) Cells were treated with media or transfected with siB5, sieIF2α, or siHVEM and then 48h later, infected with HSV-2(G) and 4h pi, cell lysates were prepared and evaluated for expression of viral ICP4 and β-actin; a representative blot from 3 independent experiments is shown (left). The blots were scanned and results are presented as odu relative to cells treated with media (mean + sd from 3 experiments). (C). Cells were grown on 96-well plates, transfected with siB5 or sieIF2α or treated with 0.1% N-9, Effectene, or untreated and then quadruplicate wells were evaluated daily for 5 days for cell viability using an MTT assay. Results are presented as percent viability relative to untreated cells and are means ± sd obtained from 2 independent experiments.

Most studies of interactions between HSV and host cell initiation factors have focused on eIF2α because of its established role in host defense. A major cellular response to viral infection (or other stress) is to inactivate eIF2α by phosphorylation. HSV overcomes this host response through the actions of at least two viral proteins, ICP34.5 and Us11, which cooperate to restore eIF2α pools [21]. While eIF2, a heterotrimer composed of three subunits, is essential for protein synthesis, the precise role eIF2α, the regulatory subunit, plays in HSV protein translation has not been established. Thus, we compared the impact of silencing eIF2α and B5 on viral infection. Transfection of CaSki cells with sieIF2α efficiently silenced its expression by ∼80%; siB5 had little or no effect on eIF2α expression (Fig. 9A). Transfection with either siB5 or sieIF2α reduced ICP4 expression as detected by Western blots of infected cellular lysates 4 h pi (Fig. 9B) and also inhibited viral plaque formation by at least one log (not shown). However, there was a marked difference in the effects of silencing on cell viability. Silencing of eIF2α reduced cell viability by ∼50% 3 days post-transfection, whereas siB5 had little effect (Fig. 9C). The cytotoxic effects observed with sieIF2α were comparable to those observed with the detergent N-9 [22]. These results suggest that B5 may provide a safer and more specific target to block HSV protein synthesis.

### B5 provides a target for prevention of primary or recurrent HSV

**Figure 10:**
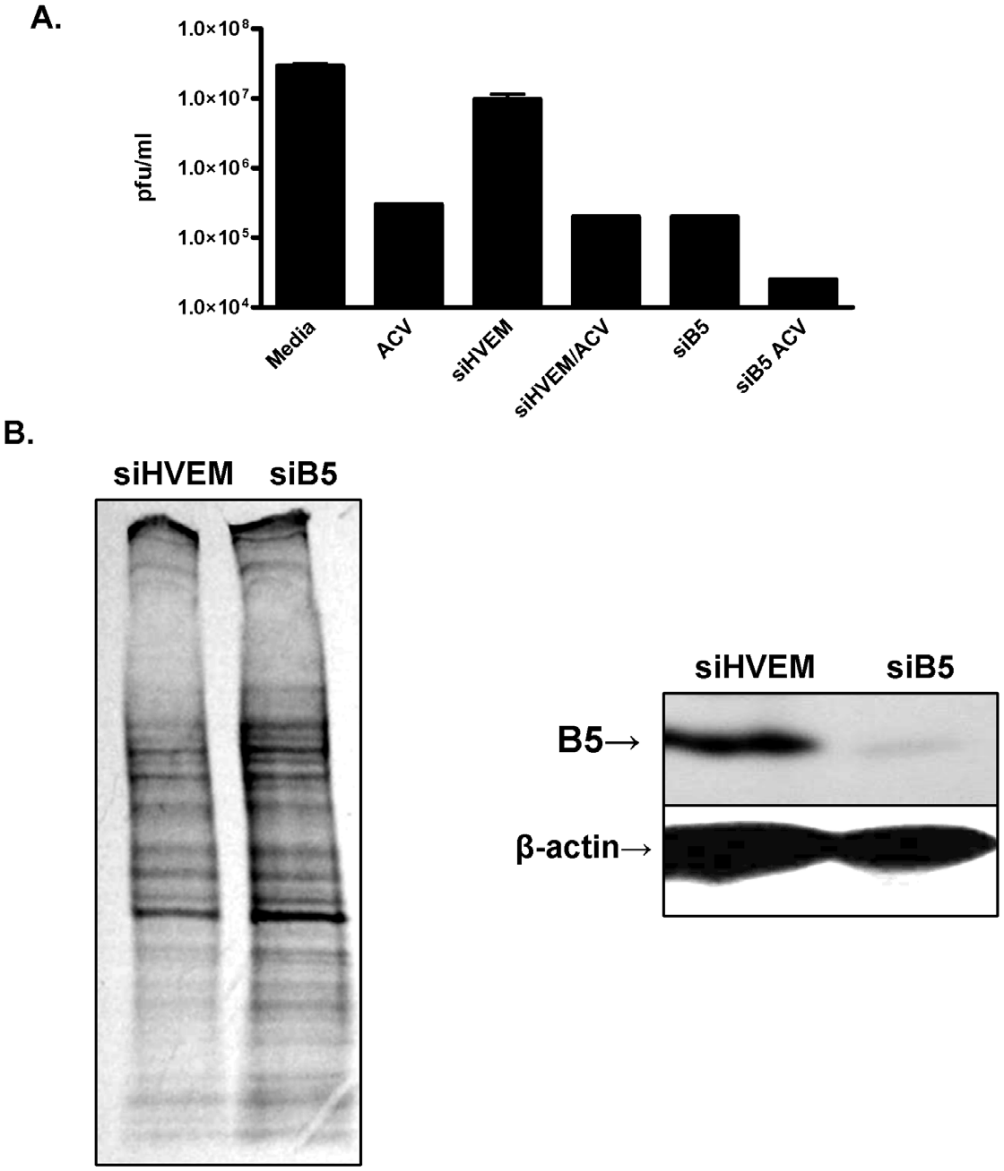
Acyclovir and B5 silencing inhibit HSV infection and silencing has little impact on host cell protein synthesis. (A). Cells were either transfected with HVEM or B5 specific siRNA or not transfected, and then infected with serial dilutions of HSV-2(G) (0.001–1000 pfu/cell) in the absence or presence of acyclovir (ACV) (100µg/ml) and 48 h pi, cells were fixed and viral plaques counted. Only wells with 20–150 pfu were counted to determine the viral titer (pfu/ml). (B). Transfected cells were metabolically labeled 72 h post-transfection and analyzed by gel electrophoresis followed by autoradiography. Results are representative of 3 independent experiments (left). Immunoblot of cells 72 h post-transfection probed with antibodies to B5 and β-actin (right).

Currently, the primary modality for treatment or suppression of HSV infections is acyclovir, which interferes with viral DNA replication. We compared the effects of acyclovir and B5 silencing on viral infection alone, or in combination. Cells were transfected with siB5 or siHVEM and then challenged with serial dilutions of HSV-2(G) in the absence or presence of 100µg/ml acyclovir and the viral plaques determined. Treatment with acyclovir or transfection with siB5 each independently reduced viral plaque formation by ∼2-logs and, when combined, resulted in ∼3-log reduction (Fig. 10A), indicating that the two interventions target different steps in the viral life cycle. To explore whether targeting of B5 could block the amplification of virus that had reactivated, we used an explant co-cultivation model. Sacral ganglia were removed from vaginally infected mice and co-cultured for two weeks with CaSki cells that had been transfected with siB5 or control siRNA; viral amplification was monitored by collecting a portion of the culture supernatants and assaying for the presence of virus by plaque assay. Little or no virus was detected in culture supernatants harvested from co-cultures of sacral ganglia and B5-silenced cells, whereas >10^4^ pfu/ml were recovered from co-cultures of ganglia with cells transfected with control siRNA in two independent experiments (not shown).

To further explore the potential safety of targeting B5 to reduce primary or recurrent HSV, the effects of B5 silencing on the overall pattern of host protein synthesis were examined by metabolic labeling the cells 72 h post-transfection. Little change in host cell protein expression was observed (Fig. 10B, left); the efficiency of silencing was confirmed by Western blots (Fig. 10B, right). These findings, coupled with the lack of cytotoxicity in B5 transfected cells (Fig. 9C) indicate that B5 is not essential for host cell protein translation and support the notion that B5 could provide a novel target for antiviral therapeutic strategies.

## Discussion

Productive HSV infection requires the translation of viral proteins and is associated with the selective inhibition of host protein synthesis. While substantial work has focused on delineating how HSV triggers the shutoff of host protein synthesis, far less is known about the molecular mechanisms that promote viral protein synthesis. The results of this study begin to address this gap. Specifically we found that B5, which has been recently identified as eIF3m, plays a key role in promoting the initial translation of HSV immediate early proteins, ICP4 and ICP0. The pivotal role B5 (eIF3m) plays in initiating the synthesis of HSV immediate early proteins may be relatively specific, as silencing of B5 had little effect on HIV, more modest effects on VSV, and little or no impact on host cellular proteins including eIF2α and nectin-1. The exact determinant that regulates which viral or cellular transcripts will interact with B5 requires further study.

Currently at least 12 eIFs, composed of at least 29 distinct subunits, have been identified [23]. The largest mammalian initiation factor, eIF3, plays an essential role in translation by binding directly to the 40S ribosomal subunit to recruit mRNAs that bear a methylated guanosine cap at the 5′-end and promote formation of the 43S preinitiation complex. Mammalian eIF3 consists of 13 non-identical subunits, eIF3a through eIF3m; subunit m was the most recently discovered [15]. The functions of the individual subunits in human cells are not yet well characterized. In the yeast *Saccharomyces cerevisiae*, eIF3 consists of a core complex of five stoichiometric subunits (eIF3a, -b, -c, -g, and -i), plus a non-stoichiometric subunit, eIF3j. In mammalian cells, the corresponding homologues also may constitute a “core” complex to which the other mammalian subunits bind and regulate eIF3 activity. The finding that silencing of B5 had little effect on cell viability and that it did not adversely impact expression of the cellular proteins studied is consistent with the notion that eIF3m is not an essential core element. However, further studies are needed.

The notion that distinct subclasses of eIF3 complexes, containing different combinations of core and non-core subunits, may regulate specific subsets of mRNA has been suggested in studies with fission yeast, where biochemically distinct eIF3 complexes defined by the PCI domain proteins eIF3e and eIF3m associate with different sets of mRNAs [24]. Tandem mass spectrometry demonstrated that eIF3m is likely to be on the periphery of the eIF3 complex, and possibly accessible to viral mRNA [15]. Additionally, time-resolved spectroscopy has shown that the B5 C-terminus forms a reversible structure that might allow it to interact with proteins, viral RNA or RNA-protein complexes [25]. Notably, a recent study found that eIF3m may also play a role in regulating caspase 9 responses to apoptotic stimuli [26].

Previous studies have explored interactions between HSV proteins and eIF4 and eIF2α. Specifically, ICP0 and ICP6 stimulate the assembly of eIF4F complexes, which facilitate subsequent viral protein synthesis [27], [28]. However, these interactions must occur downstream of the interactions between HSV immediate early mRNA and eIF3m (B5). The ability of HSV to antagonize eIF2α phosphorylation, which would prevent formation of the 80S ribosomal complex and thus block viral protein synthesis, has been extensively studied. Results of the current studies are consistent with the role of eIF2α in viral protein translation, as silencing reduced HSV gene expression and plaque formation (Fig. 9). However, silencing of eIF2α is toxic to cells and thus would not provide a target for antiviral therapies.

The observation that both acyclovir and siB5 markedly inhibit HSV infection by different mechanisms supports the notion that targeting of B5 could provide a novel strategy to prevent primary and recurrent HSV infections. This is critical, not only because of the morbidity associated with HSV infection itself, but because of the synergy between HSV and HIV. In recently completed clinical trials, suppressive acyclovir therapy failed to reduce the risk for HIV acquisition [29]. One possible explanation for this comes from recent work showing the persistence of activated immune cells in sequential biopsies of HSV-2 lesions from patients on suppressive antiviral therapy [30]. Acyclovir inhibits viral replication, but does not prevent viral reactivation or block viral protein expression, and therefore may not prevent the recruitment and activation of HSV-specific T cells. Results obtained in this work suggest that B5 silencing might prevent the amplification of reactivating virus and possibly limit the recruitment and activation of T cells, which may serve as HIV targets.

The discovery that B5 plays a key role in promoting the synthesis of HSV immediate early proteins was serendipitous. The original observation that B5 may contribute to HSV infection came from studies with porcine cells that are resistant to HSV entry and did not address the translation of viral proteins. Transfection of the porcine cells with a human cDNA expressing B5 rendered the cells fully susceptible to HSV [11]. However, in ongoing work, we found that B5 does not play a direct role in mediating entry into the porcine cells, but acts indirectly and modulates the expression of porcine nectin, rendering it accessible to bind viral gD (work in progress). Notably, we observed no changes in expression of human nectin-1 following B5 silencing. In conclusion, the current studies demonstrate that B5 (eiF3m) plays a major role in promoting the translation of HSV immediate early proteins and may be a novel target for prevention strategies.

## Materials and Methods

### Cells and Viruses

CaSki cells (human cervical epithelial cell line) were obtained from American Tissue Culture Collection (ATCC CRL 1550) and were passaged in DMEM supplemented with 10% fetal bovine serum. Jurkat-Tat-CCR5 cells, a T-cell line transfected with the HIV-1 *tat* gene and the CCR5 coreceptor, were cultured in complete RPMI supplemented with 250 µg/ml hygromycin B (for Tat selection) and 500 µg/ml geneticin (for CCR5 selection) as previously described [31]. HSV-2(G), HSV-1(KOS), and HSV-1(KVP26GFP), which contains a green fluorescent protein (GFP)–VP26 fusion protein [16] were prepared from infected Vero cell cultures (ATCC CCL81) and viral stocks were stored at −80°C. HIV-1_BaL_ was grown in PM-1 cells and stored at −80°C after filtration through 0.2 µm filters (Millipore, MA). VSV-Indiana was grown on Vero cells.

### Viral labeling and purification

To label viral envelopes, Vero cells were infected with wild-type viruses or KVP26GFP virus (∼0.001 pfu/cell) and after 48 h, the infected cells were lysed and then incubated with lypophilic tracers DiO or DiI (1 µM) (Molecular Probes, Invitrogen Valencia, CA) for 10 min at room temperature before purification on sucrose gradients [4].

### siRNA and transfections

Cells were transfected with 100pmol/well (12-well plates) of the indicated siRNA by using the Effectene transfection reagent (QIAGEN, Valencia, CA) as described previously [17]. Nectin-1 siRNA (ID214887,12154,111208) B5 siRNA (ID122597,122596,122595), HVEM siRNA (ID13772,111368,214894), eIF2α siRNA (ID33496,125660,125659) and control siRNA (Silencer negative control siRNA#1, AM4636) were purchased from Applied Biosystems (Ambion; Austin, TX) Cells were analyzed for protein expression by preparing Western blots of cell lysates and for gene expression by qRT-PCR (see below).

### Viral infection and VP16 assays

Transfected or control cells were infected with serial dilutions of virus for 1 h at 37°C, inoculum removed, and cells overlaid with medium in the absence or presence of acyclovir (100µg/ml) (American Pharmaceutical Partners, Schaumburg, IL) for 48 h. Plaques were counted by immunoassay using an anti–human IgG antibody peroxidase conjugate (Calbiochem) [32]. For synchronous infections, cells were precooled and exposed to virus at 4°C for 4 h to allow binding. Unbound virus was removed by washing, and the cells were transferred to 37°C to allow viral penetration for 45 min. For VSV, CaSki cells that had been transfected with siRNA 48 h earlier were infected with serial dilutions of VSV, overlaid with 1% methylcellulose, fixed after 24 h with methanol, and plaques counted after staining with Giemsa.

To examine transport of VP16 to the nucleus, nuclear extracts were prepared 45 min after infection and analyzed by Western blots as described previously [10]. To examine viral protein or gene expression, whole cell lysates were prepared and protein expression examined by Western blots or RNA extracted for qRT-PCR (below). In select experiments, cycloheximide (100µg/ml, C-7698, Sigma-Aldrich) was added to the overlay media for the entire time of infection as a control for inhibition of protein synthesis.

### Total RNA extraction and real-time quantitative RT-PCR (qRT-PCR)

Total RNA was extracted using an Absolutely RNA Miniprep kit (Stratagene, La Jolla, CA). RNA (500ng) was reverse transcribed with the High Capacity cDNA Reverse Transcription kit (Applied Biosystems, Foster City, CA). Real-time PCR amplification was performed using an Applied Biosystems 7900HT Real-Time PCR System and analyzed using the sequence detector software. The reactions were performed using the TaqMan® Gene Expression Master Mix and the probes for human nectin-1 (PVRL1) (Hs00161050_m1), HVEM (TNFRSF14) (Hs00187058_m1), B5 (GA17) (Hs00272235_m1), interferon alpha (IFNA1) (Hs00256882_s1) and RPLPO (4310879E) were obtained from Applied Biosystems. Quantification was normalized against the number of RPLPO transcripts in the same RNA extracts.

To evaluate expression of HSV genes, primer and probe sequences were designed using Primer Express (Applied Biosystems) software and synthesized by Applied Biosystems as 20× Mix. The HSV-2 primers and probe used were as follows: 1) HSV2 ICP0: forward primer 5′-GTGCATGAAGACCTGGATTCC-3′; reverse primer 5′-GGTCACGCCCACTATCAGGTA-3′; and probe 5′-FAM-TTGCGCAACACGTGTCCCCTG-TAMRA-3′; 2) HSV-2 ICP4: forward primer 5′-GCGAGCTGCGGTTCGT-3′; reverse primer 5′-GCCACGCGCAGGTC-3′; and probe 5′-FAM-CAGGCGCATCAGCACC-TAMRA-3′; 3) HSV-2 gB: forward primer 5′-CTCGCCGAGCTGTACGT-3′; reverse primer 5′-CGGGCGTGGCATTCC-3′; and probe 5′-FAM-CTCCCGCATGTACTCC-TAMRA-3′.

### Antibodies for Western blots and confocal microscopy

Antibodies and dilutions were as follows: anti-nectin murine monoclonal antibodies (mAbs), 1∶500 (sc-21722, Santa Cruz Biotechnology, Santa Cruz, CA); anti-VP16 mAb, 1∶500 (sc7545; Santa Cruz Biotechnology); anti-ICP27 mAb, 1∶2000 (ab31631, Abcam, Cambrige, Ma), anti-ICP4 mAb 1∶500 (P1101, Virusys Corporation, Sykesville,MD), anti-ICP0 mAb, 1∶500 (H1A027-100,Virusys); anti-gB mAb, 1∶500 (HA056-100, Virusys); anti-gD mAb, 1∶1000 (P1103, Virusys), anti-β-actin mAb, 1∶5000 (A-5441; Sigma-Aldrich, St. Louis, MO), anti-HVEM mAb 1∶200 (ab374820, Abcam), anti-B5 rabbit polyclonal, 1∶500 (ab69842, Abcam), anti-eIF2α 1∶500 (ab5369), anti-histone H1, 1∶1000 (sc-8030; Santa Cruz Biotechnology),anti-golgin97, 1∶500 (A-21270, Invitrogen), anti-RPLP0 1∶500 (ab88872, Abcam) and anti-PABP mAb, 1∶500 (sc-32318; Santa Cruz Biotechnology). The secondary antibodies for Western blots were horseradish peroxidase-conjugated goat anti-mouse, 1∶1000 (170–5047, Bio-Rad, Hercules, CA), or goat anti-rabbit 1∶1000 (170–5046, Bio-Rad).

### Confocal Microscopy

Cells were grown on glass coverslips in 12-well plates, transfected with siRNA as described above, and, if indicated, 24 or 48 h post-transfections, synchronously infected with DiI-labeled HSV-1(KVP26GFP). To label plasma membranes, the cells were stained for 30 min with EZ-Link sulfosuccinimidobiotin (EZ-Link Sulfo-NHS-Biotin) reagent (0.1 mM; Pierce Chemical, Rockford, IL), which reacts with primary amines on cell-surface proteins before infection, fixed with 4% paraformaldehyde solution (Electron Microscopy, Hatfield, PA) at the indicated times pi, and the biotinylated cells were reacted with Alexa Fluor 350-conjugated streptavidin (1∶1000 dilution; S11225, S11249; Invitrogen, Carlsbad, CA). Images were acquired by laser confocal microscope ZeissLive/DuoScan equipped with an oil immersion objective 63×1.4. Images were captured in an optical slice of ∼0.5 µm with appropriate filters, Alexa Fluor 488, and GFP were excited using the 488-nm line of a krypton/argon laser and viewed with a 505- to 530-nm band pass µm. AlexaFluor 360 were excited with 405-nm diode laser and collected with 420-to 475 nm filter, Alexa 546 were excited using 543-nm helium/neon laser and collected with a 575- to 655 filter. All images were captured using the multitrack mode of the microscope to decrease cross talk of fluorescent signals. Z-sections were captured in an optical slice of 0.5 µm.

Live image microscopy was performed to examine calcium signaling in response to HSV. Cells were grown in glass bottom culture dishes 35 mm (product no. P35G-1.5-10-C; MatTek corporation, Ashland, MA) and labeled with EZ-Link and 350-conjugated streptavidin (1∶1000 dilution; S11225, S11249; Invitrogen). The cells were then loaded with Calcium Crimson (C-3018, 2.5µg/ml, Invitrogen) for 4 h at 4°C, and infected with DiO-labeled virus for 4 h at 4°C. Cells were washed three times with PBS to remove extracellular Ca^2+^ and unbound virus, overlaid with 25 mM HEPES buffer, and placed into a temperature-regulated 37°C environmental chamber in a ZeissLive/DuoScan confocal microscope fitted with a 100×1.4 oil objective. Images were acquired 10 min after the dishes were placed in the chamber. Image analysis was conducted using the LSM confocal software package (Carl Zeiss, Inc.) and quantification of intensity staining with image J software (National Institutes of Health, Bethesda, MD). Three-dimensional (3-D) images were generated using the Volocity 4 confocal software (Improvision, Lexington, MA).

### RNA-protein complexes and immunoprecipitation

CaSki cells were grown on 25cm^2^ flasks, transfected with siRNA at 800pM per flask using the Effectene transfection reagent (QIAGEN, Valencia, CA) and 48h post-transfection, the cells were infected with HSV2(G) (moi 2 pfu/cell). Three, 5 or 7 h pi, the cells were treated with 0.04% of methanol-free formaldehyde (RT 15680, Electron Microscopy sciences, Hatfield, PA) to cross-link RNA-protein complexes or with PBS as a control and immunoprecipitated with a polyclonal rabbit anti-B5 antibody in RIPA buffer (Sigma-Aldrich) supplemented with complete protease inhibitors (Roche Diagnostics) as described [33]. The RNA was purified from the supernatant and the precipitated complexes (pellet) and analyzed by qRT-PCR using primers for ICP4, ICP0, gB or RPLPO. Quantifications were normalized per input RNA. Enrichment was defined as the ratio of normalized number of viral transcripts in each fraction relative to transcripts in the input (non-immunoprecipitated). Immunoblots were also prepared and analyzed for viral proteins or RPLPO.

### Polyadenylated RNA extraction

Subconfluent CaSki cells were transfected with 800pmol/well (T25 dish) of siHVEM or siB5 and then 48 h post-transfection were infected with HSV2(G) (3 pfu per cell). Three hours pi, cells were harvested and dissolved into iso-hi-pH buffer [10mM Tris pH 8.4, 140mM NaCl, 1.5mM MgCl_2_, 0.5% NP-40] supplemented with 10mM VRC (S1402S, New England BioLabs, Ipswich, MA). Nuclei were pelleted for 10 min at 1500 rpm, washed twice in iso-hi-pH and once in iso-hi-pH supplemented with 0.1 volume of Tween-deoxycholate solution, pelleted through a 2.2 M sucrose pad for 30 min at 16,000 rpm, and washed again in iso-hi-pH. To examine the purity of isolated cellular fractions, Western blots were performed using golgin97 and histone H1 as markers of cytoplasm and nuclear fractions, respectively. Total RNA was purified from the total cellular lysates, cytoplasmic and nuclear fractions by TRIzol, and polyadenylated mRNA was subsequently isolated from the total RNA preparations using Absolutely mRNA™ purification kit (400806, Stratagene) and visualized by formaldehyde denaturing gels. Expression of ICP4 was detected by qRT-PCR.

### Isolation of polysomes

Subconfluent CaSki cells were transfected with 800pmol/well (T25 dish) of siHVEM or siB5 and then 48 h post-transfection were left uninfected or were infected with HSV2(G) (3 pfu per cell) for 3 h. Both the uninfected and infected cells were trypsinized and the cell pellets were rinsed twice with ice-cold PBS and resuspended in gradient buffer [50mM Tris-HCL (pH 7.5), 80mM KCl, 5mM MgOAc, 2 mM DTT, 1% sucrose] containing 1 unit/µl of RNase inhibitor (RNAsin, N2611, Promega, Madison, WI) and supplemented with complete protease inhibitors (Roche). Cells were lysed by sonication, subjected to centrifugation (16,000 rpm for 10 min at 4°C) and then layered on top of an 11-ml linear 10–50% sucrose gradient. Samples were centrifuged at 27,500 rpm in a Beckman SW41 ultracentrifuge rotor for 4 h. Gradients were fractionated from the bottom (1 ml fractions) and analyzed for PABP, B5 or ICP4 by Western blotting. Alternatively, the RNA in each fraction was purified with TRIzol LS reagent (Life Technologies, Gaithersburg, MD) followed by phenol-chloroform extraction and ethanol precipitation and dissolved into 10 µl of RNAase free water. The fractions were assayed for absorbance at 254 nm with a Nano Drop spectrophotometer. To evaluate for ICP4 transcripts, 2µl of RNA recovered from each fraction were subjected to reverse transcription and then the cDNA was amplified with primers specific for ICP4 or GAPDH (ICP4: forward 5′-GTACGTCAGCGGGGAGCC-3′ and reverse 5′-GTCGCCGTCGAAGCCCTC-3′; GADPH: forward 5′-GCATGGCCTTCCGTGTCCC C-3′ and reverse 5′-TGGCAGGGACTCCCCAGCAG-3′. DNA was visualized by electrophoresis in 1% agarose.

### HIV infection and p24 assay

Jurkat-TAT-CCR5 cells were infected with HIV-1 BaL (4ng of p24 per 10^4^ cells) for 2 hours and then washed and overlaid with RPMI growth media. Culture supernatants were collected and viral replication was assessed using a p24 enzyme-linked immunosorbent assay (ELISA; p24 antigen capture assay kit; NCI-Frederick Cancer Research and Development Centre, AIDS Vaccine Program).

### S^35^–methionine labeling of cellular proteins

CaSki cells (60% confluent 6-well plates) were transfected with siHVEM and siB5 RNA at 160pM per well using Effectene transfection reagent in DMEM without L-methionine and L-cystine (21013-024, GIBCO), supplemented with 0.3mCi/ml of L-S^35^-methionine (6009, 1175Ci/mmol, NE). The cells were harvested 72 h post-transfection and analyzed in a 4–20% gradient polyacrylamide gel(161–1105, Bio-Rad), followed by autoradiography using HyBlot CL film (E3018, Denville Scientific Inc.).
